# Topical Donepezil for Cholinergic Modulation in Chronic Wound Repair

**DOI:** 10.1111/wrr.70211

**Published:** 2026-09-08

**Authors:** Hui‐Qi Qu, Charlly Kao, Hakon Hakonarson

**Affiliations:** ^1^ The Center for Applied Genomics Children's Hospital of Philadelphia Philadelphia Pennsylvania USA; ^2^ Department of Pediatrics The Perelman School of Medicine, University of Pennsylvania Philadelphia Pennsylvania USA; ^3^ Division of Human Genetics Children's Hospital of Philadelphia Philadelphia Pennsylvania USA; ^4^ Division of Pulmonary and Sleep Medicine Children's Hospital of Philadelphia Philadelphia Pennsylvania USA; ^5^ Faculty of Medicine University of Iceland Reykjavik Iceland

**Keywords:** acetylcholinesterase inhibition, chronic wounds, donepezil, nitric oxide, non‐neuronal cholinergic system, topical dermal delivery, α7‐nAChR

## Abstract

Chronic wounds result from combined defects in vascular perfusion, inflammatory resolution, and epithelial repair. The skin's non‐neuronal cholinergic system (NNCS), formed by keratinocytes, endothelial cells, fibroblasts, and immune cells that synthesise and respond to acetylcholine (ACh), helps coordinate these processes through muscarinic and nicotinic receptors. Inhibition of acetylcholinesterase (AChE) increases local ACh concentrations and may engage two key pathways supported by preclinical data: M3 muscarinic receptor (M3 mAChR)‐endothelial nitric oxide synthase (eNOS)‐nitric oxide (NO)‐mediated vasodilation, and α7 nicotinic acetylcholine receptor (α7‐nAChR)‐mediated suppression of pro‐inflammatory cytokines. Donepezil is a reversible, selective AChE inhibitor with physicochemical properties compatible with dermal administration. Low‐dose or microneedle dermal delivery has produced dermal exposure with limited systemic uptake in animal and ex vivo skin studies. In preclinical diabetic wound models, nicotinic receptor activation accelerated healing, reduced inflammatory signalling, and improved control of bacterial burden. We hypothesise that topical donepezil formulated for localised dermal delivery could restore cholinergic signalling within the wound microenvironment by increasing local ACh concentrations. This approach may complement metabolic therapies that support arginine‐NO coupling and redox balance. Controlled pilot studies should assess local cutaneous pharmacodynamics, perfusion responses, and wound‐closure outcomes.

## 1 Chronic Wounds

Chronic wounds are defined as skin defects that fail to proceed through the normal phases of healing, haemostasis, inflammation, proliferation and remodelling, within an expected timeframe, and thus persist in a non‐resolving state [1]. In the context of a growing global burden of metabolic disease, vascular dysfunction and aging populations, chronic wounds have become one of medicine's most persistent and costly problems. The annual cost of chronic wound management in developed health systems runs into tens of billions of dollars, and global wound‐care expenditure was recently estimated at USD 148 billion in 2022 [2].

Among the major chronic wound phenotypes, diabetic foot ulcers (DFUs) represent the prototypical and most prevalent form. A recent review estimated that DFUs affect approximately 18.6 million people worldwide each year [3]. A systematic meta‐analysis found the global prevalence of DFUs among adults with diabetes to be ~6.3% (95% CI: 5.4%–7.3%) with higher rates in men than women [4]. The pathogenesis of DFUs is multifactorial: peripheral neuropathy reduces protective sensation; mixed macro‐ and microvascular ischemia impede perfusion and nutrient delivery; repeated micro‐trauma in a neuropathic foot; and microbial biofilm formation and infection sustain inflammation and block granulation. For example, a substantial proportion of DFU patients have peripheral arterial disease contributing to ischemia, and approximately 50% of DFUs become infected [5]. Because DFUs frequently precede lower‐limb amputations (up to ~80% of non‐traumatic amputations occur in people with DFUs) and carry a five‐year mortality rate in the order of ~30% or higher, they disproportionately contribute to morbidity, mortality and health‐system cost [3, 6].

Current management of DFUs and other chronic wounds is rooted in a multifaceted approach: off‐loading (in DFUs) or compression/bandaging (in venous ulcers), vascular assessment with revascularisation where indicated, rigorous infection and biofilm management including debridement and antimicrobials, moisture balance and wound‐bed preparation, and optimisation of systemic factors such as glycemic control, nutrition, renal/cardiac comorbidities and smoking cessation. Adjunctive therapies, such as negative pressure wound therapy, cellular/tissue‐based products (skin substitutes), and advanced dressings, have shown incremental benefit in selected populations but do not reliably achieve closure or prevent recurrence in many patients [7].

Nevertheless, several key barriers persist. Microvascular dysfunction remains a major bottleneck: even after macro‐vascular revascularisation, capillary perfusion and oxygen delivery may remain inadequate [8]. Persistent inflammation and oxidative stress lead to endothelial uncoupling, impaired nitric oxide (NO) signalling, and delayed granulation. Biofilm‐mediated infection sustains chronic inflammatory activation, further delaying healing [9]. Recurrence rates remain high (e.g., > 40% at 1 year, ~65% at 5 years in DFUs) despite initial closure [3, 4]. Patient adherence challenges (off‐loading, compression and wound‐care regimen), socioeconomic disparities, and the fact that most therapies act downstream (structural and metabolic support) rather than upstream (regenerative signalling) all contribute to the ongoing gap.

Given this mechanistic landscape, ischemia/hypoxia, non‐resolving inflammation, impaired epithelial migration/repair, and persistent microbial burden, therapeutic strategies that restore endogenous regulatory signalling (rather than merely providing structural support) are especially compelling. Increasing evidence indicates that chronic diabetic and ischemic wounds also exhibit disrupted non‐neuronal cholinergic system (NNCS) function characterised by increased acetylcholinesterase activity [10, 11, 12] with reduced M3‐eNOS‐NO vasodilatory signalling and impaired perfusion [13, 14, 15], diminished α7‐JAK2/STAT3 activation with heightened inflammatory cytokine output (TNF‐α and IL‐6) [16, 17], and reduced keratinocyte migration at the wound edge [18, 19]. These cholinergic deficits intersect directly with the vascular, inflammatory and epithelial bottlenecks described above and thus provide rationale for targeted cholinergic‐modulation approaches to chronic wounds.

## 2 The NNCS in Skin Homeostasis and Wound Repair

The skin possesses a fully operational NNCS that mirrors key features of neuronal ACh signalling yet functions autonomously to regulate cutaneous homeostasis, inflammation, and repair [20]. Keratinocytes, fibroblasts, endothelial cells, and immune cells synthesise, release, and degrade acetylcholine (ACh), forming a local paracrine‐autocrine loop that influences vascular tone, immune balance, and epithelial dynamics [10]. ACh synthesis is catalysed by choline acetyltransferase (ChAT), released through constitutive, Ca^2+^‐dependent, and non‐vesicular mechanisms, and degraded by acetylcholinesterase (AChE) and butyrylcholinesterase (BChE). The resulting ‘cholinergic tone’ shapes vasoregulation, epithelial integrity, and immune quiescence [11, 12].

### 2.1 Muscarinic Signalling and Vascular‐Epithelial Regulation

Binding of ACh to muscarinic M3 receptors (M3R) on endothelial cells triggers Ca^2+^‐dependent eNOS activation and nitric oxide (NO) production, promoting microvascular vasodilation [15]. Complementary prostacyclin and EDHF pathways provide NO‐independent vasodilatory routes [21]. Endothelial cells can also release ACh in response to mechanical activation by shear stress, forming an autocrine loop that sustains vasodilatory signalling during flow changes and tissue injury [13, 14].

In keratinocytes and fibroblasts, M3R signalling influences cytoskeletal organisation, junctional communication, and extracellular‐matrix remodelling, processes central to wound‐edge migration and closure [19]. During tissue repair, activation of these muscarinic pathways supports perfusion of the wound margin and the metabolic demands of angiogenesis [13, 22].

### 2.2 Nicotinic Signalling and Immunomodulation

Nicotinic ACh receptors (nAChRs), particularly α7‐nAChR, modulate inflammatory balance within the skin. α7 activation on macrophages and dendritic cells inhibits TNF‐α, IL‐1β and HMGB1 release via JAK2/STAT3 and NF‐κB suppression, constituting the canonical cholinergic anti‐inflammatory pathway [17]. This signalling facilitates the transition of macrophages from M1 to M2 phenotypes and contributes to resolution of inflammation during tissue repair. Additional roles for α7‐nAChR include regulation of immune‐cell migration and coordinated inflammatory clearance [16]. Selective α7 agonism enhances matrix deposition and fibroblast recruitment in experimental wound‐healing models [23].

### 2.3 Keratinocyte Cholinergic Function and Barrier Integrity

Keratinocytes express muscarinic and nicotinic receptors and synthesise ACh de novo [18]. Cholinergic signalling regulates keratinocyte proliferation, migration, adhesion, and differentiation, all essential for re‐epithelialisation [10]. Local ACh release at wound margins stimulates lamellipodial extension and integrin‐mediated attachment, while nicotinic and muscarinic inputs coordinate collective migration [18, 19]. Beyond migration, cholinergic pathways modulate filaggrin and loricrin expression and calcium‐dependent differentiation gradients, supporting barrier reformation [19, 24].

### 2.4 Integrated Cholinergic Axis in Cutaneous Repair

Across vascular, immune, and epithelial compartments, the NNCS integrates ACh‐dependent vasoregulation, inflammatory control, and epithelial remodelling into a coordinated repair program [20]. The M3‐eNOS‐NO pathway supports microvascular flow and angiogenesis; the α7‐nAChR pathway restrains excessive cytokine signalling and supports reparative macrophage transitions; and keratinocyte receptor cross‐talk directs wound‐edge migration and barrier formation. These interacting nodes form a unified cholinergic repair axis that maintains perfusion, immune balance, redox control and epithelial closure.

## 3 Cutaneous Wound Modulation by Acetylcholinesterase Inhibition

**FIGURE 1:**
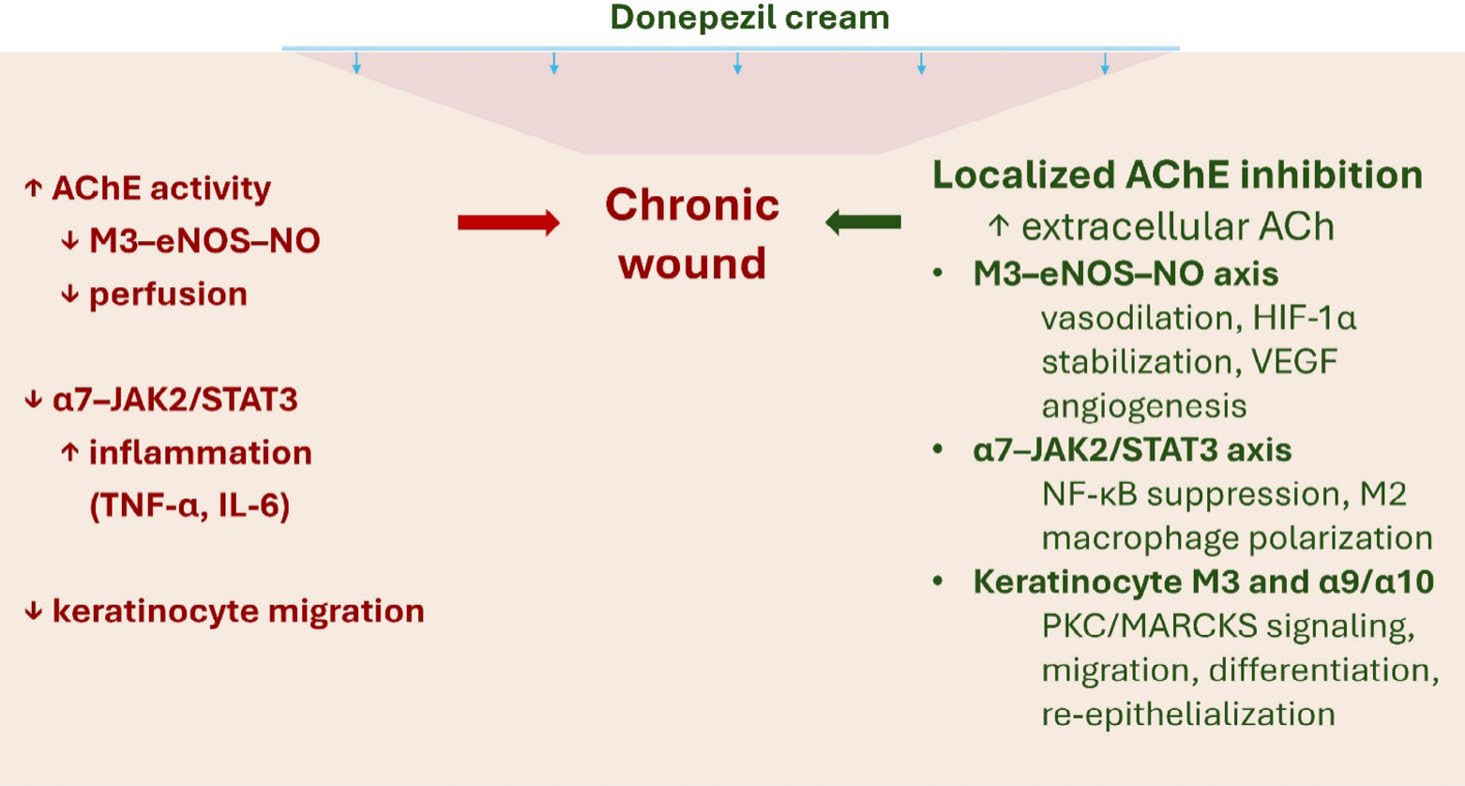
Localised acetylcholinesterase inhibition in chronic wound repair using AT‐004. Chronic diabetic or ischemic wounds exhibit impaired perfusion, persistent inflammation, and stalled epithelial migration due to disrupted NNCS signalling. Topical AT‐004 (donepezil) is applied to the wound surface, where it locally inhibits AChE and increases extracellular acetylcholine. Elevated ACh reactivates muscarinic M3 receptors on endothelial cells to drive eNOS‐NO‐dependent vasodilation, HIF‐1α stabilisation, and angiogenesis, and stimulates α7‐nAChR on macrophages to suppress NF‐κB‐mediated cytokine release and promote M2 polarisation. Concurrent activation of muscarinic and nicotinic receptors on keratinocytes enhances migration and differentiation, accelerating re‐epithelialisation. Together, these pathways restore vascular, immune and epithelial balance in the chronic wound microenvironment. Local topical or microneedle delivery confines AChE inhibition to the wound bed, enhancing cholinergic signalling while minimising systemic exposure.

Convergent evidence from skin enzymology, ischemic tissue models, and diabetic wound systems indicates that acetylcholinesterase inhibitor (AChEI) can restore coordinated vascular, immune and epithelial signalling impaired in chronic wounds. The available data define a mechanistic framework in which AChEI enhances perfusion, accelerates inflammation resolution, promotes epithelial regeneration and supports angiogenesis through integrated cholinergic pathways (Figure 1).

### 3.1 Cutaneous Cholinesterase Distribution and Activity Shifts

Human skin expresses both AChE and BChE, which together regulate extracellular ACh levels produced by keratinocytes, endothelial cells, fibroblasts and immune cells [10, 12]. AChE is concentrated in keratinocytes and perivascular endothelium, controlling epithelial differentiation and microvascular tone. In inflammatory and diabetic settings, AChE activity may become imbalanced, with evidence of altered AChE and BChE regulation [11]. Residual AChE activity at wound edges could therefore represent a pharmacologic target.

### 3.2 Mechanistic Engagement of AChEI in the Wound Environment

Prolongation of ACh signalling by AChEI stabilises M3 receptor‐dependent eNOS‐NO output under hypoxic or ischemic conditions, improving perfusion and oxygen delivery [13, 14, 15]. By prolonging ACh availability, AChEI is expected to amplify the α7‐nAChR‐dependent anti‐inflammatory pathway described above, thereby facilitating inflammatory resolution [16, 17]. In diabetic models, selective α7 agonism with PNU‐282987 accelerated closure and inhibited AGE‐induced inflammatory signalling, while topical nicotinic stimulation decreased bacterial survival and TLR2 activation [25, 26]. These data suggest that AChEI amplifies both vascular and immune components of the wound microenvironment.

AChEI enhances keratinocyte motility and differentiation by prolonging cholinergic signalling at the wound surface. Sustained ACh exposure increases PKC and MARCKS phosphorylation, promoting actin remodelling and integrin recycling necessary for collective cell migration [18]. Concurrently, calcium‐dependent muscarinic signalling supports epidermal differentiation and expression of barrier proteins such as filaggrin and loricrin [19]. In chronic wounds, where cholinergic signalling is typically diminished, this action may restore epithelial advancement and barrier reformation.

### 3.3 Vascular and Redox Coupling During AChE Inhibition

Nitric oxide generated by M3‐eNOS signalling stabilises HIF‐1α through S‐nitrosylation‐mediated inhibition of prolyl hydroxylases, enhancing VEGF expression and angiogenesis [27, 28]. These NO‐linked mechanisms predict increased VEGF signalling, microvascular density, and reduced inflammatory tone under AChE inhibition, restoring vascular‐redox coupling [29].

### 3.4 Topical Dermal Delivery Feasibility

Microneedle (MN) and 3D‐printed hybrid platforms incorporating donepezil provide sustained intradermal delivery, adequate mechanical strength, and controlled release profiles in both rats and ex vivo porcine skin [30, 31]. Earlier topical AChEIs, such as physostigmine and neostigmine, induced localised vasodilation and sudomotor activation without systemic toxicity [12]. Donepezil in the formulation AT‐004 is directly targeted at topical dermal delivery with retention of active drug in the epidermis and dermis [20, 32]. These findings validate the feasibility of localised cholinergic augmentation in the cutaneous environment.

### 3.5 Genetic and Transcriptomic Modifiers of Cholinergic Responsiveness

Emerging genomic evidence indicates that the skin's NNCS is subject to inter‐individual genetic variation. The *CHRNA7* gene, which encodes the α7‐nicotinic acetylcholine receptor, is dynamically up‐regulated during wound healing in mouse skin [33]. The human‐specific fusion gene *CHRFAM7A* modulates α7‐nAChR activity and exhibits structural and copy‐number variation among individuals [34, 35, 36]. Cholinergic gene expression in epidermal and immune compartments changes under stress or injury, suggesting plasticity of the local acetylcholine‐regulating network [37]. Together, these findings highlight a precision‐medicine opportunity: patients with genetically or transcriptionally reduced NNCS activity may respond preferentially to localised acetylcholinesterase inhibition. Future pilot studies could integrate genotyping or wound‐edge transcriptomic profiling of NNCS components to stratify therapeutic response to localised donepezil or related cholinergic modulators.

Population‐scale genomic data indicate that NNCS is under strong purifying selection, supporting its causal indispensability in tissue repair. In gnomAD v4.0 [38], key catalytic and receptor genes show marked depletion of predicted loss‐of‐function (pLoF) variants: *ACHE* [observed/expected (o/e) = 0.37; pLI = 0.81], *CHRM3* (o/e = 0.25; pLI = 1.0), *CHRNA7* (o/e = 0.66; pLI = 0; missense o/e = 0.51), and *SLC5A7* (o/e = 0.30; pLI = 1.0) all display constraint comparable to essential signalling genes. More moderate constraint in *CHAT* (o/e = 0.79) and tolerance in *BCHE* (o/e = 0.94) reflect redundant enzymatic buffering, while the constrained human‐specific *CHRFAM7A* (o/e = 0.27; pLI = 0.76) suggests selective maintenance of α7‐receptor dosage. Across the NNCS, an evolutionary gradient of constraint emerges, stringent conservation of catalytic, receptor, and transport components with progressively greater tolerance in modulatory elements such as *SLURP1*, *CHRNA9*, and *CHRNA10*. This architecture indicates that acetylcholine synthesis, degradation, and receptor signalling are evolutionarily nonredundant, whereas peripheral ligands and accessory proteins retain adaptive flexibility for tissue‐specific tuning. Such hierarchical constraint implies evolutionary safeguarding of cholinergic tone essential for vascular and epithelial homeostasis. The scarcity of common functional alleles thus explains the absence of genome‐wide or Mendelian associations with chronic wounds, indicating that NNCS function is important and evolutionarily constrained.

### 3.6 Classes of AChEIs

AChEIs comprise several pharmacologic subclasses that differ in reversibility, enzyme selectivity and physicochemical properties, distinctions that carry direct implications for cholinergic modulation in the wound microenvironment (Table 1). Topical agents such as physostigmine and neostigmine demonstrated that peripheral AChE inhibition can elicit local cholinergic responses in skin with limited systemic spillover [12]. Their hydrophilicity and short duration of action, however, limit their suitability for chronic wound settings, where sustained augmentation of cholinergic tone is needed to support prolonged deficits in perfusion, inflammatory resolution and epithelial migration. These agents nevertheless provide important proof‐of‐concept that peripheral AChE inhibition is pharmacologically actionable within the cutaneous compartment.

**TABLE 1:** Major acetylcholinesterase inhibitors relevant to cutaneous cholinergic modulation.

Agent	Reversibility/enzyme selectivity	Considerations for chronic wounds
Donepezil	Reversible; highly selective for AChE (> 1000‐fold vs. BChE) [39]	AT‐004 is a lipophilic tertiary amine designed for epidermal/dermal retention. Other formulations are intended for transdermal penetration with established human pharmacokinetics [40, 41]; microneedle‐compatible [30, 31]. Their selective AChE inhibition aligns with targeted NNCS modulation (M3‐eNOS‐NO, α7‐nAChR).
Rivastigmine	Pseudo‐irreversible carbamate; dual AChE/BChE inhibition	Sustained enzyme inactivation may decrease ability to titrate local exposure; BChE inhibition may unpredictably affect keratinocyte differentiation and immune balance [10, 11]; transdermal systems exist but offer less mechanistic specificity for NNCS‐targeted wound therapy.
Galantamine	Reversible AChE inhibition; allosteric potentiator of nAChRs	Additional nicotinic receptor potentiation complicates interpretation of α7‐nAChR‐mediated anti‐inflammatory signalling [16, 17, 42]; lacks validated dermal or transdermal delivery systems; less predictable specificity in chronic wound environments.
Physostigmine/Neostigmine	Reversible; limited AChE versus BChE selectivity	Hydrophilicity limits sustained dermal penetration; short‐acting but demonstrate proof‐of‐concept for peripheral cholinergic activation [12]; useful mechanistically but suboptimal for chronic application requiring stable cholinergic support.

Centrally acting tertiary‐amine inhibitors, including donepezil, rivastigmine and galantamine, were originally developed for Alzheimer's disease but possess physicochemical characteristics that also favour dermal penetration. These agents differ meaningfully in their selectivity and binding kinetics. Rivastigmine produces pseudo‐irreversible inhibition of both AChE and butyrylcholinesterase (BChE), forming a carbamylated intermediate that persists for many hours. Because BChE participates in keratinocyte differentiation, immune regulation, and local cholinergic buffering [10, 11, 12], dual AChE/BChE inhibition may produce less predictable effects in chronic wounds in which cholinesterase activity is already altered. Galantamine is fully reversible but also serves as an allosteric potentiator of nicotinic receptors, which may be beneficial but complicates interpretation of α7‐nAChR‐mediated anti‐inflammatory signalling [16, 17, 42]. In contrast, donepezil exhibits high potency and marked selectivity for AChE over BChE [39], thereby enabling targeted prolongation of endogenous ACh signalling within muscarinic and nicotinic pathways most relevant to wound repair [15, 18].

From a delivery perspective, donepezil is an AChEI and the AT‐004 formulation of donepezil under development by Arctic Therapeutics is a well‐characterised molecule intended for topical dermal delivery with retention of the active ingredient in the epidermis and dermis of the skin for optimal skin delivery and retention [20, 32]. In contrast, transdermal system in humans for treatment of Alzheimer's disease demonstrates predictable absorption, acceptable tolerability, and plasma exposure comparable to oral administration [40, 41]. This positions the AT‐004 formulation of donepezil as an agent with unusually favourable translational readiness for localised topical dermal delivery around wound margins.

These comparative considerations support the prioritisation of donepezil for NNCS‐targeted wound therapeutics. Its reversible, highly selective inhibition of AChE aligns with mechanistic goals of amplifying the cholinergic vasodilatory and anti‐inflammatory pathways implicated in wound repair, while avoiding perturbation of BChE‐dependent processes or additional receptor‐level agonism. Its potency, lipophilicity, and long half‐life and unique topical dermal delivery with stable local exposure while minimising systemic uptake make AT‐004 a highly competitive therapeutic opportunity for treating inflammatory skin diseases and wound healing. Taken together, these properties distinguish donepezil from other AChEIs and provide a coherent rationale for advancing it as the lead candidate for localised cholinergic modulation in chronic wound repair.

## 4 Donepezil as a Cholinergic Modulator

Donepezil is a reversible, selective acetylcholinesterase inhibitor with a pharmacologic profile suitable for localised cutaneous use. Donepezil is highly potent and long‐acting. Even small systemic absorption can lead to unintended cholinergic side effects (e.g., bradycardia, nausea and dizziness). This pharmacodynamic sensitivity underscores the importance of delivery approaches that restrict systemic exposure while maintaining local efficacy. Nonetheless, its potency, bioavailability, and safety record support investigation as a dermal modulator of cholinergic signalling in chronic wound repair (Table 2). The therapeutic rationale centers on augmenting local acetylcholine tone to promote vascular, inflammatory and epithelial recovery mechanisms without engaging central cholinergic pathways.

**TABLE 2:** Experimental and mechanistic evidence supporting donepezil in tissue repair.

Domain/model	Key findings	Evidence type	Level	Quality^a^	Representative sources
Pharmacokinetics and delivery feasibility	Unlike the transdermal patch (10–20 mg day^−1^) achieved systemic exposure comparable to oral dosing, AT‐004 topical application demonstrates sustained intradermal retention with limited systemic exposure.	Human PK (Phase I); Preclinical device PK	2b/5	B/C	[30, 31, 40]
Vascular/angiogenic mechanisms	↑ VEGF, ↑ eNOS phosphorylation, ↑ microvascular density; improved perfusion and flap survival via M_3_‐eNOS‐NO and HIF‐1α/VEGF activation.	Animal/mechanistic	5	C	[29, 43, 44]
Anti‐inflammatory/cytoprotective actions	↓ TNF‐α, IL‐6, oxidative stress; activation of α7‐nAChR‐JAK2/STAT3; reduced apoptosis and enhanced viability in ischemic models.	Animal/mechanistic	5	C	[45, 46]
Epithelial/keratinocyte response (in vitro)	AChE inhibition (physostigmine, neostigmine) → ↑ Ca^2+^ signalling, PKC activation, keratinocyte migration and differentiation.	Cell culture	5	C	[18]
Cutaneous wound and ischemia models (indirect & direct evidence)	Topical dermal application ofAChE inhibition achieves therapeutic skin exposure, and nicotinic receptor activation in diabetic wound models enhances perfusion, reduces inflammation, and accelerates closure.	Human PK & preclinical mechanistic	2b/5	B/C	[12, 25, 26, 47]
Bone/osteogenic effects (adverse context)	Post‐operative donepezil ↓ bone regeneration and implant osseointegration due to excessive muscarinic Ca^2+^ signalling.	Animal	5	C	[48]
Safety/tolerability (topical/systemic)	Transdermal trials: mild transient erythema; no systemic cholinergic toxicity at therapeutic dose.	Clinical PK	2b	B	[40]

^a^ Evidence levels: 1 = Meta‐analysis; 2a = Randomised clinical trial; 2b = Non‐randomised or Phase I–II clinical study (e.g., PK/tolerability); 3 = Observational; 4 = Case series; 5 = Preclinical/Mechanistic. Quality: A = Strong; B = Moderate; C = Preliminary.

### 4.1 Pharmacologic Characteristics and Selectivity

Donepezil exhibits nanomolar potency for AChE and over 1000‐fold selectivity versus BChE [39]. Such selectivity allows highly targeted modulation of acetylcholinesterase while limiting off‐target cholinergic effects in peripheral tissues. While the compound's tertiary amine structure and moderate lipophilicity (logP≈4.1) favour dermal penetration [49], the AT‐004 formulation of donepezil targets topical dermal delivery and epidermal/dermal skin retention. These physicochemical traits facilitate diffusion within and across the stratum corneum. With an elimination half‐life of approximately 70 h, donepezil supports sustained cholinergic activity intradermally at low systemic exposure, whereas donepezil patches and other formulations necessitate careful dose and surface‐area control to avoid accumulation and systemic exposure. Partial enzyme occupancy of 50%–70% appears sufficient to elevate tissue ACh and engage vascular and immune pathways without receptor desensitisation, indicating that submaximal inhibition is adequate to achieve biological activity while maintaining an acceptable safety margin.

### 4.2 Dermal and Intradermal Delivery Systems

Transdermal donepezil has been validated in human studies. A single patch delivering 10–20 mg per day achieved plasma concentrations equivalent to oral dosing (Cmax 5–20 ng/mL) with minimal irritation [40], confirming consistent pharmacokinetics via the cutaneous route. A once‐weekly patch system (ADLARITY) provided bioequivalence to oral 10 mg with lower peak‐trough variation and improved tolerability [41, 50]. Efforts to reduce plasma exposure are expected to reduce adverse events and improve adherence. Approved weekly systems deliver nominal doses of 5 mg/day or 10 mg/day, establishing a regulatory precedent for sustained transdermal dosing of donepezil, whereas AT‐004, currently under development, delivers the active ingredient intradermally with retention in the epidermis/dermis layers of the skin.

### 4.3 Delivery‐Platform Considerations for Localised Cholinergic Activation

Achieving localised acetylcholinesterase inhibition while minimising systemic donepezil exposure is a central translational requirement for cutaneous cholinergic modulation. Because donepezil is highly potent, moderately lipophilic, and long‐acting, the choice of delivery system strongly favours formulations that remain on the surface, such as creams, hydrogels that offer straightforward application and advantage in the presence of wound topography, barrier integrity and local hydration. These factors can lead to uneven dermal deposition and variable proximity to target vascular and immune cell populations. Platforms that introduce drug past the outer barrier allow more predictable localisation.

Several emerging systems increase the precision of local exposure through environmental responsiveness or structural confinement. Smart hydrogels, ultradeformable lipid vesicles and electrospun nanofiber dressings can alter release characteristics in response to moisture, pH, or mechanical strain, enabling closer alignment between drug availability and wound physiology [51, 52, 53]. Device‐assisted methods such as iontophoresis and fractional‐laser microporation further restrict drug entry to defined sites, offering focal increases in permeability without altering intact surrounding tissue [54, 55].

In situ‐forming injectable depots, photocrosslinkable hydrogels, thermoresponsive sol–gel systems and lipid‐polymer hybrid nanoparticle matrices represent the highest level of spatial control. These systems can be deposited directly along wound margins, where they harden or self‐assemble into conformal reservoirs that release drug within a narrowly defined volume [56, 57, 58]. This geometry‐matching property is particularly advantageous for chronic wounds with irregular or undermined edges. Ex vivo preconditioning of autologous keratinocyte‐fibroblast sheets provides an alternative mode of localisation, in which cholinergic pathway activation is embedded within the graft rather than delivered as free drug [59].

Collectively, these delivery systems illustrate a broad range of options for tailoring dermal exposure in chronic wounds, as summarised in Table 3. However, most platforms either lack predictable intradermal retention or require specialised procedures that limit practical use. Microneedle‐based systems, for example, although capable of depositing drug directly into the dermis, were not designed to retain highly potent, lipophilic, and long half‐life molecules such as donepezil within a confined cutaneous compartment. Dissolving or coated microneedles typically release their payload rapidly and can increase the risk of systemic absorption if the applied dose, needle density or skin hydration varies. These systems also require device‐based application and are difficult to adapt to irregular wound margins, which limits their feasibility in routine wound care. In contrast, AT‐004 cream is specifically formulated to localise donepezil within the epidermis and dermis, producing controlled intradermal AChE inhibition while minimising systemic uptake. This predictable retention profile aligns directly with the therapeutic objective of localised cholinergic activation and supports AT‐004 as the most practical and clinically translatable approach among the delivery strategies reviewed in Table 3.

**TABLE 3:** Comparative delivery routes for localised dermal cholinergic activation with donepezil.

Delivery route	Localisation	Systemic risk	Feasibility	Notes (donepezil‐specific)
AT‐004 topical cream (optimised for localised dermal residency)	Moderate‐high (epidermis/dermis)	Low	High	Vehicle composition (pH, viscosity and solubility balance) is tuned to favour epidermal and dermal residency of donepezil, supporting localised AChE inhibition while limiting systemic penetration.
Conventional cream/hydrogel	Moderate	Moderate	High	Simple periwound application; donepezil's lipophilicity implies variable flux and less control.
Matrix patch	High	Low	Medium	Controlled diffusion suits donepezil's long half‐life, but area must be limited to avoid accumulation [40].
Reservoir patch	High	Low	Medium	Precise release of potent donepezil; less flexible for irregular wound surfaces [40].
Dissolving microneedle (MN)	Very high	Minimal	High	Delivers microgram‐scale donepezil directly into the dermis [30]. Dissolution occurs rapidly, and because MNs lack mechanisms for dermal retention, lipophilic small molecules such as donepezil may diffuse systemically after delivery.
Coated/Hollow MN	High	Low	Medium	Rapid dermal dosing of donepezil possible, but needs sustained system to maintain exposure [31].
Hybrid MN‐patch	Very high	Minimal	Medium	Combines instantaneous MN dermal entry with patch‐mediated controlled release [31]. Provides focal delivery but does not ensure sustained dermal retention of lipophilic drugs. Geometry limits use on irregular wound margins.
Smart hydrogel/nanofiber mat	Variable	Low	Emerging	Responsive to wound hydration/pH [52], adaptive donepezil flux, still experimental.
Ethosomal/ultradeformable vesicle systems	High	Low‐Moderate	Emerging	Lipid‐ethanol vesicles enhance skin retention of lipophilic drugs like donepezil, reducing systemic escape [51, 60].
Electrospun drug‐loaded nanofiber dressing	Very high	Minimal	Emerging‐medium	Nanofiber scaffold applied to wound margin can load microgram donepezil, releasing locally with negligible systemic exposure [53, 61].
Iontophoresis‐enabled patch (Donepezil·HCl)	Tunable (restricted area)	Low‐moderate	Medium	Low‐intensity current drives charged donepezil salt into skin in pulses, time/area control limits systemic uptake [54].
Laser‐assisted drug delivery (ablative fractional laser + donepezil topical)	High (micro‐channels)	Moderate	Specialised/Emerging	Ablative microchannels enhance topical donepezil entry into dermis, very focal, but systemic risk rises if coverage large [55].
Injectable dermal depot platforms (photopolymerizable/thermoresponsive sol–gel/lipid‐polymer hybrid nanoparticles)	Very high (periwound dermal depot)	Minimal	Medium	Injectable dermal depots create highly localised reservoirs for microgram‐scale donepezil release directly in periwound dermis [56, 57, 58]. These platforms support sustained cholinergic activation with negligible systemic uptake while conforming to irregular wound margins.
Autologous tissue‐engineered skin/cell sheets preconditioned ex vivo with donepezil	Very high (graft only)	Minimal	Experimental/Low	Keratinocyte‐fibroblast sheets [59] pre‐treated with low‐dose donepezil then washed allow highly localised cholinergic modulation with minimal system‐wide exposure.

### 4.4 Pharmacokinetics and Exposure Control

In transdermal pharmacology, absorption depends on both the molecule's physicochemical properties and the characteristics of the delivery system [62]. Drugs with molecular weights below ≈500 Da, moderate lipophilicity (logP≈1–4), and limited hydrogen‐bonding capacity penetrate the stratum corneum most efficiently, criteria largely met by current commercially available forms of donepezil. The molecule therefore possesses intrinsic suitability for percutaneous transport. Systemic uptake follows Fick's law of diffusion and can be adjusted by varying concentration, matrix composition, surface area, and skin hydration. Slower, localised release can be achieved through rate‐controlling membranes, reduced drug loading, or biodegradable microneedle polymers such as hyaluronic acid, polyvinylpyrrolidone, or polycaprolactone, which dissolve gradually over hours to days [63]. The use of occlusive dressings or penetration enhancers can be modulated according to whether systemic or local exposure is desired. In this venue, AT‐004 was developed to maintain therapeutic epidermal/dermal concentrations of donepezil while avoiding excessive plasma levels.

Given its long half‐life noted above, surface area and release rate require careful control to prevent accumulation. Even modest changes in applied dose or patch dimensions can produce measurable systemic differences over time. Pharmacodynamic biomarkers such as transcutaneous oxygen tension (TcPO_2_), laser‐Doppler perfusion, and wound‐exudate cytokine profiles offer objective means to verify local cholinergic activation, with pharmacokinetic confirmation that systemic exposure remains low [64]. AT‐004, currently in active clinical development, maintains this therapeutic window with reproducible control.

### 4.5 Preclinical and Translational Evidence

In rodent limb‐ischemia and skin‐flap models, donepezil increased eNOS phosphorylation, VEGF expression, and microvascular density while reducing TNF‐α and IL‐6, consistent with activation of the HIF‐1α‐VEGF pathway and improved redox balance [43, 44]. These findings link cholinergic modulation to angiogenic and anti‐inflammatory mechanisms directly relevant to wound repair.

Synergistic angiogenesis was observed when donepezil was combined with sildenafil [43], indicating possible benefit from multimodal vascular stimulation. AChEI therapy also upregulated Nrf2 and Akt signalling, preserved mitochondrial integrity, and improved survival during ischemia–reperfusion injury [44, 45]. Collectively, the evidence supports a cytoprotective effect in hypoxic or ischemic tissue environments and a broader role for donepezil in restoring microvascular homeostasis.

### 4.6 Safety Considerations and Tissue‐State Dependence

Systemic donepezil produces dose‐related cholinergic adverse events at high plasma levels, including bradycardia, gastrointestinal disturbance and dizziness [39]. Accordingly, dermal delivery systems must be designed and monitored with explicit attention to systemic spillover. In this regard, AT‐004 is specifically designed for epidermal/dermal retention and minimal systemic absorption. While microneedle delivery reduces systemic uptake, it requires surveillance for irritation or edema, necessitating concurrent evaluation of cutaneous tolerability. Excessive M3‐receptor stimulation can enhance endothelial permeability and inflammatory activation [65], underscoring the need for precise dose regulation. During later remodelling or ossification phases, prolonged muscarinic activation may interfere with mineralisation through Runx2 and BMP‐Smad signalling, consistent with reports of delayed bone healing [48, 66, 67]. Temporal control of therapy is thus essential—application should be confined to early and mid‐healing stages and tapered as tissue matures.

### 4.7 Translational Perspective and Clinical Endpoints

Localised donepezil delivery represents a precision approach to restoring cholinergic tone in chronic wounds. By acting directly on dermal neurovascular and immune signalling, this strategy integrates pharmacology with the biology of impaired healing. Pilot clinical studies should assess changes in microvascular perfusion (TcPO_2_, laser‐Doppler), inflammatory cytokines (TNF‐α and IL‐6), CRP and epithelial closure rates as primary endpoints [64, 68]. These outcome measures connect mechanistic activity with clinically relevant repair metrics.

Systemic pharmacokinetic data must confirm sub‐therapeutic plasma levels, as this will determine regulatory acceptance of localised cholinergic therapy. A Phase 2 trial using oral donepezil for refractory diabetic wounds [69] highlights the translational interest in this mechanism, and forthcoming results may help define dose–response relationships applicable to dermal delivery. Further clinical development will depend on controlled exposure, biomarker validation, and integration of pharmacodynamic endpoints with healing outcomes to establish both safety and proof of concept in chronic wound management.

## 5 Integration With Metabolic Support Therapies

Chronic wounds typically combine hypoperfusion, oxidative stress, and persistent inflammation with stalled epithelialisation. Metabolic therapy, centered on arginine, glutamine, ascorbate and zinc, addresses substrate and redox constraints but does not directly target cholinergic receptors or acetylcholinesterase activity.

### 5.1 Substrate and Redox Pillars

#### 5.1.1 Arginine and Endothelial Coupling

Arginine, the obligate substrate for eNOS, supports vasodilation when eNOS remains properly coupled [70]. Under oxidative stress or tetrahydrobiopterin (BH_4_) deficiency, eNOS becomes uncoupled, reducing NO output and increasing superoxide generation [71]. Nutritional arginine supplementation can help restore NO production capacity, yet endothelial M3 receptor stimulation remains important to activate eNOS in situ [14, 22].

#### 5.1.2 Glutamine and Antioxidant Defence

Glutamine fuels rapidly dividing and repairing cells and is a biochemical precursor for glutathione; human studies show modulation of antioxidant defences (e.g., increased GPx activity) with supplementation [72], although whole‐blood GSH may not rise in healthy volunteers [73]. Restoration of glutamine stores thus supports both cellular energy metabolism and oxidative homeostasis during tissue repair.

#### 5.1.3 Ascorbate and Collagen Maturation

Ascorbate (vitamin C) acts as a cofactor for prolyl‐ and lysyl‐hydroxylases, enzymes essential for hydroxylation of collagen triple helices and subsequent crosslinking [74]. Adequate vitamin C availability ensures structural integrity of granulation tissue and tensile strength of the wound bed, functions often compromised in deficiency states [75, 76].

#### 5.1.4 Zinc and Matrix Remodelling

Zinc is indispensable for numerous DNA/RNA polymerases, metalloproteases, and transcription factors that regulate immune competence, re‐epithelialisation and extracellular‐matrix remodelling. Deficiency impairs keratinocyte migration and delays closure [77]. Supplemental zinc corrects these deficits and supports balanced inflammation and epithelial repair.

### 5.2 Clinical Signal for Nutrition

In malnourished adults with stage II‐IV pressure injuries, an oral formula enriched with arginine, zinc and antioxidants significantly improved wound healing versus isocaloric control in a randomised controlled trial [78]. Subsequent analyses and international guidelines confirmed these findings, recommending high‐protein, arginine‐, zinc‐ and antioxidant‐enriched nutritional support for patients with pressure injuries or other chronic wounds [79]. These results validate metabolic correction as a necessary adjunct for perfusion and signalling control.

### 5.3 Why Pair With Cholinergic Modulation

Metabolic therapy restores the capacity for repair, supplying substrates, cofactors and redox balance, whereas cholinergic modulation restores the command, re‐engaging the regulatory pathways that drive perfusion, inflammatory resolution, and epithelial closure. These two domains converge mechanistically across vascular, immune, and epithelial compartments. In the vascular axis, cholinergic vasoregulation through the M3‐eNOS‐NO pathway operates synergistically with arginine‐supported nitric oxide synthesis [14, 22]. In the immune compartment, α7‐nAChR‐mediated anti‐inflammatory signalling complements glutathione‐dependent redox control [16]. Within the epithelium, muscarinic receptor activation promotes keratinocyte migration and differentiation, effects that are further supported by ascorbate‐ and zinc‐dependent enzymatic processes [18].

Together, metabolic support and cholinergic enhancement may yield synergistic benefits, including increased microperfusion, reduced oxidative tone, improved matrix quality and accelerated re‐epithelialisation, providing a coherent rationale for their integrated application in chronic wound repair.

### 5.4 Translational Implications

A pragmatic translational framework places metabolic optimisation (adequate protein/energy intake with arginine, glutamine, ascorbate and zinc per trial and guideline evidence) as the foundational step, followed by localised cholinergic enhancement to activate the endothelial and immune pathways necessary for wound closure. In this sequence, metabolic therapy restores the biochemical capacity for nitric oxide generation, redox balance, and matrix synthesis, while localised acetylcholinesterase inhibition provides the upstream cholinergic signal that engages the M_3_‐eNOS‐NO and α7‐JAK2/STAT3 pathways. To evaluate this combined strategy, early translational endpoints should incorporate microvascular and inflammatory biomarkers, such as transcutaneous oxygen tension (TcPO_2_), laser‐Doppler perfusion, and wound‐exudate cytokine panels, alongside planimetric assessment of closure rates. These measures can verify local pharmacodynamic engagement while maintaining safety oversight for both cholinergic adverse events and micronutrient excess. Although mechanistic support for cholinergic regulation of eNOS activity and redox homeostasis is robust, and nutrition trials demonstrate moderate clinical benefit, direct human studies that integrate NNCS‐targeted therapy with metabolic support remain lacking. Addressing this gap will require phased pilot studies that link dermal delivery, receptor‐level responses and microvascular perfusion to healing outcomes, providing the evidence base for a combined cholinergic‐metabolic therapeutic paradigm in chronic wound repair (Table 4).

**TABLE 4:** Translational roadmap for localised cholinergic‐metabolic integration in chronic wound repair.

Translational domain	Current evidence	Critical gap	Actionable next step/study design	Representative sources
Mechanistic Integration (M_3_‐eNOS‐NO & α7‐nAChR axes)	Preclinical vascular and anti‐inflammatory activation demonstrated.	Limited human perfusion data linking cholinergic activation to closure outcomes.	Early‐phase study using TcPO_2_ and laser‐Doppler flowmetry (LDF) as pharmacodynamic markers of local cholinergic engagement.	[64, 68]
Pharmacokinetic feasibility (transdermal/microneedle)	Human patch PK validated; rat microneedle PK promising.	No dermal pharmacodynamic correlation or exposure‐response data.	Combine local PK (patch or microneedle) with cytokine (TNF‐α, IL‐6, and CRP) and perfusion readouts to define local vs. systemic exposure. Pilot PK‐PD study using low‐dose microneedle or topical patch to characterise dermal donepezil exposure, perfusion (LDF/TcPO_2_), and cytokine modulation.	[30, 40, 44]
Receptor‐level specificity	Variable α7‐nAChR and M_3_ expression in wound compartments.	Lack of receptor‐resolved human data.	Stratify biopsy or scRNA‐seq analyses by receptor subtype; correlate expression with healing trajectories.	[16]
Safety/phase‐specific modulation	Osseous inhibition reported in rats.	Risk during bone remodelling or late proliferative phases.	Define dermal dosing margins; exclude patients with active fracture or osteogenic repair.	[40, 48]
Integrative therapeutic design (metabolic + cholinergic)	Nutritional arginine‐zinc‐antioxidant formula improves closure in RCTs.	No combined metabolic + cholinergic human studies.	Pilot Phase 1b/2a trial: donepezil patch ± nutrient formula on ischemic/diabetic ulcers; endpoints = TcPO_2_, LDF, cytokines, planimetric closure.	[44, 78]

## 6 Conclusion and Clinical Outlook

Donepezil should be regarded as a regulatory modulator of wound biology, rather than a uniformly reparative agent. Its actions appear phase‐dependent, with potential benefit during periods dominated by ischemia or inflammation, and a possibility of adverse effects if exposure extends into the later phases of ossification or tissue remodelling. Accordingly, clinical translation should prioritise topical dermal delivery approaches with skin retention that ensures verified local target engagement while maintaining tightly controlled systemic exposure. Early pilot studies should aim to demonstrate consistent, quantifiable local pharmacodynamic effects under carefully regulated exposure conditions and to define tolerability thresholds appropriate for subsequent trial phases. Clinical application should correspond to the early to mid stages of healing, with deliberate avoidance of treatment during active bone remodelling or ossification phases [48]. Finally, combining localised cholinergic augmentation with metabolic optimisation represents a conceptually synergistic strategy and warrants evaluation within pragmatic clinical trial designs [16, 44, 78].

## Funding

This work was supported by sponsored research funds from Arctic Therapeutics ehf., Institutional Development Funds from The Children's Hospital of Philadelphia to the Center for Applied Genomics, and the Children's Hospital of Philadelphia Endowed Chair in Genomic Research (HH).

## Ethics Statement

The authors have nothing to report.

## Conflicts of Interest

Dr. Hakonarson has a method of use patent treating wound healing with AChEIs. Drs. Hakonarson and Kao have a new method of use patent application pending for treating inflammatory skin diseases and are co‐founders of Arctic Therapeutics ehf. The authors declare no other potential conflicts of interest with respect to the research, authorship, and/or publication of this article.

## Data Availability

Data sharing not applicable to this article as no datasets were generated or analysed during the current study.
